# Long-term CPAP treatment partially improves the link between cardiac vagal influence and delta sleep

**DOI:** 10.1186/1471-2466-13-29

**Published:** 2013-04-30

**Authors:** Fabrice Jurysta, Chantal Kempenaers, Jean-Pol Lanquart, André Noseda, Philippe van de Borne, Paul Linkowski

**Affiliations:** 1 Sleep Laboratory and Laboratory of Psychiatric Research, Department of Psychiatry, Erasme Academic Hospital - ULB, Brussels, Belgium; 2 Chest Department, Erasme Academic Hospital-ULB, Brussels, Belgium; 3 Department of Cardiology and Hypertension Clinic, Erasme Academic Hospital - ULB, Brussels, Belgium

## Abstract

**Background:**

Continuous positive airway pressure (CPAP) treatment improves the risk of cardiovascular events in patients suffering from severe sleep apnea-hypopnea syndrome (SAHS) but its effect on the link between delta power band that is related to deep sleep and the relative cardiac vagal component of heart rate variability, HF_nu_ of HRV, is unknown. Therefore, we tested the hypothesis that CPAP restores the link between cardiac autonomic activity and delta sleep across the night.

**Methods:**

Eight patients suffering from severe SAHS before and after 4 ± 3 years of nasal CPAP treatment were matched with fourteen healthy controls. Sleep EEG and ECG were analysed to obtain spectral sleep and HRV components. Coherence analysis was applied between HF_nu_ and delta power bands across the first three sleep cycles.

**Results:**

Sleep characteristics and spectral HRV components were similar between untreated patients, treated patients and controls, with the exception of decreased Rapid Eye Movement duration in untreated patients. Coherence and gain values between HF_nu_ and delta EEG variability were decreased in untreated patients while gain values normalized in treated patients. In patients before and during long-term CPAP treatment, phase shift and delay between modifications in HF_nu_ and delta EEG variability did not differ from controls but were not different from zero. In healthy men, changes in cardiac vagal activity appeared 9 ± 7 minutes before modifications in delta sleep.

**Conclusions:**

Long-term nasal CPAP restored, in severe SAHS, the information between cardiovascular and sleep brainstem structures by increasing gain, but did not improve its tightness or time shift.

## Background

Increased cardiovascular events reportedly occur in the early morning hours [1,2]. Interaction between cardiac autonomic activity and sleep was studied to explain this increased incidence [3-5].

The causal link between sleep apnea-hypopnea syndrome (SAHS) and cardiovascular morbidity has remained controversial for many years [6,7] but there is now strong evidence that sleep apnea is an independent risk factor for cardiovascular disease [8]. Imbalance of cardiac sympatho-vagal activity was found in SAHS but exact physiological process involved in cardiac dysregulation remained unclear. Apnea induces bursts in peripheral sympathetic activity [9] and raises vagal activity [10]. Patients suffering from SAHS showed decreased relative cardiac vagal influence [11-13] and had inadequate sympatho-vagal responses to the environment [14] or to sleep stage changes [15,16].

In patients suffering from SAHS, continuous positive airway pressure (CPAP) treatment improved sleep architecture [17,18], cardiovascular indices [19,20] and other comorbidities such as metabolic syndrome, cardiovascular diseases or asthma [21-24]. Only a few studies have evaluated the impact of long–term CPAP treatment on the cardiac sympatho-vagal balance. The majority showed an increase in vagal predominance according to the spectral components of heart rate variability (HRV) after CPAP treatment [19,20]. However, other studies reported conflicting results [25,26]. Some authors also studied the evolution of cardiac sympathetic and vagal HRV components across sleep stages in severely apneic patients treated by CPAP [27,28]. Results were limited to the first night of treatment.

No studies have investigated the impact of long-term CPAP treatment on spectral HRV components across sleep stages, or the evolution of the link between HRV and deep sleep across the night in severely apneic patients treated by CPAP, in comparison to healthy controls.

HRV components have been studied across sleep stages in healthy young and middle-aged men. All authors found that heart rate decreased during non-Rapid Eye Movement (NREM) sleep in comparison to Rapid Eye Movement (REM) sleep or wakefulness [3,4,29]. Cardiac vagal activity, which is related to the spectral component of High Frequency (HF) of HRV and which oscillates at the respiratory frequency, was found to increase during NREM sleep, however it decreased during REM sleep and wakefulness [5,30-32]. Low frequency power (LF) of HRV is related to the sympathetic influence within the cardiac sympatho-vagal balance [30] although some controversy exists on this point [33,34]. These spectral HRV components are expressed in absolute units (in millisecondes^2^) but when expressed in normalized units (nu) they may reflect more accurately the relative influence of one system versus the other on HRV [34]. As HF_nu_ is defined as HF/(HF + LF), HF_nu_ corresponds to 1-LF_nu_. An increase in HF_nu_ is proportionally associated to a decrease in LF_nu_ and conversely. HF_nu_ is also considered as a better reflection of the relative cardiac vagal influence than absolute HF power [30,34,35]. Moreover, LF/HF is also used to measure the influence of sympathetic or vagal activity on the cardiac sympatho-vagal balance [30,34,35].

Spectral analysis can be also applied to EEG signal to define five specific frequency power bands that are delta, theta, alpha, sigma and beta power bands. Each of them has a specific cyclic alternating pattern throughout the night and the maximum of each sleep EEG frequency power band is specifically related to sleep stages [36-39].

Therefore, the relationship between cardiac vagal activity and sleep EEG power bands has been investigated [40-43]. The link between normalized HF HRV and delta power bands, of which the maximum was associated with sleep stages 3 and 4 of NREM sleep [39], was considered a stronger link compared to relationships between HF_nu_ and the different normalized sleep EEG power band [42]. Moreover, modifications in HF_nu_ preceded modifications in the normalized delta power band by 9 ± 7 to 12 ± 5 minutes in healthy middle-aged and young men, respectively [42,43]. Severe SAHS particularly altered the link between cardiac vagal influence, HF_nu_, and delta sleep EEG by decreasing coherence and gain values and by altering the phase shift or the delay between occurrence of modifications in both signals [16].

As CPAP treatment could improve the cardiac sympatho-vagal activity, as well as sleep architecture, in patients suffering from severe SAHS, we tested the hypothesis that the altered link between the relative cardiac vagal influence and delta sleep should be improved by CPAP treatment in severely apneic patients.

We assessed the link between normalized HF HRV and delta power bands throughout the night in severely apneic patients before and after at least one year of nasal CPAP (nCPAP) treatment compared with healthy matched controls.

## Methods

### Subjects

Patients suffering from severe SAHS and treated by nasal CPAP during at least one year were selected retrospectively, provided they did not develop any cardiovascular disease or a mental or neurological disorder. They had been admitted in the Sleep Laboratory for two consecutive nights to diagnose SAHS, and to exclude other sleep disorders, such as parasomnia. Subsequently, recordings were performed at least one year after all adjustments of CPAP treatment were done. Adjustments included a sleep apnea-hypopnea index (AHI) ≤ 10 events/hour and an absence of side effects reported by patients as skin irritation, dry throat, cough. Patients were recommended to maintain CPAP during a minimum of 5 hours every night. Moreover, CPAP machine documented the mean use duration.

Apnea was scored when complete breathing cessation was longer than 10 seconds. Hypopnea was noted if ventilation was reduced to less than 50% of the pre-hypopnea level and associated with a decrease in arterial O_2_ saturation greater than 3% or with arousal. Apnea was defined as obstructive, central or mixed and reported as the apnea-hypopnea index (AHI) per hour based on the ratio of apnea to total sleep time (TST). SAHS was considered severe if the apnea-hypopnea index was greater than 30 events/hour [44].

During each hospitalization, sitting systolic and diastolic blood pressures (SBP and DBP) were measured by a mercury sphygmomanometer in standardized conditions. Mean SBP and DBP were obtained for each patient. Physical examination as well as blood and urinary analyses were performed to exclude somatic pathologies.

Patients were matched for age and gender with healthy controls. Healthy men were recorded during three successive nights. The first night was used to provide accommodation to the study procedure and exclude sleep pathologies. Psychiatric and somatic disorders were also excluded. Mean SBP and DBP were calculated.

Participants were not allowed to take any illicit drugs, medication or over-the-counter (OCT) substances. Caffeine consumption was limited to 400 mg/day until 6 PM. Alcohol was tolerated for very small quantities until 6 PM. Two apneic patients and one control regularly smoked more than 20 cigarettes/day.

Participants and controls were told to go to sleep at approximately 22:30 hours and woke spontaneously in the morning. Sleeping during the day was not allowed.

Controls received a detailed description and demonstration of the procedure and signed an informed consent form. The local ethics committee of the Erasme Academic Hospital approved the study protocol.

### Recordings

For untreated patients, sleep EEG and ECG of the second night were used for analysis. For controls, recording the second or the third night was used, choosing the recording without artifacts. Artifacts were defined as electrical parasites on recordings, insertion of QRS complexes in EEG signals or deviation of EEG signals from the baseline. If neither the second nor the third night showed artifacts, one of them was randomly selected for analyses. For CPAP treated patients, the night of the second hospitalization to check CPAP treatment was used for analysis. Therefore, we analysed ECG and EEG recordings of the same patients before and during nasal CPAP treatment.

Analyzed EEG and ECG signals were obtained from a 19-channel digital polygraph (Brainnet, Medatec, Brussels, Belgium) where EEG electrodes were Cz-Ax (Ax is a mastoid reference) and ECG electrodes were positioned in the fifth intercostal space and the mid-clavicular line (V4) or horizontally even with V4 in the mid-axillary line (V6). Polygraph was composed of three EEG (Fz-Ax, Cz-Ax, Oz-Ax), two electrooculograms, one submental electromyogram, ECG with one derivation (V4 or V6), a pulse-oximetry (Biox 3740, Ohmeda, Louisville, CO) to detect oxyhemoglobin saturation, oro-nasal thermistors (Infinity, Sleepmate Technologies, Midlothian, VA), thoracic and abdominal piezo-electric sensor straps to detect respiratory movements (Resp-EZ, Sleepmate Technologies, Midlothian, VA), and ankle piezo-electric movements strain gauges to detect periodic leg movements (Moving images, Sleepmate Technologies, Midlothian, VA).

Signals were filtered and sampled at 200 Hz. Data were digitized with a 12 bit analog-to-digital converter and recorded in a digital format that we developed (Endymion, 1993–2012, Sleep laboratory, Erasme Academic Hospital – ULB) to be read and stored in the EDF file format [45]. EEG and ECG were stored at 100 and 200 Hz, respectively. ECG was upsampled at 400 Hz to obtain a maximum of precision for RR-intervals (RRI) measurement. RRI is defined as the duration of two successive R waves of the QRS complex on sleep ECG.

### Data analysis

Using the Endymion software, each 20-second epoch of the sleep EEG was visually scored in sleep stages (1 to 4, REM and wakefulness) in accordance with Rechtschaffen and Kales criteria [46]. Fast Fourier transform was applied to each five second window and results were then averaged every 20 seconds to obtain one point of spectral power every 20 seconds. The spectral power was scaled so that the power of a 50 μV digitized sine wave was 1250 (μV)^2^. The frequency limits of delta EEG power band were 0,5 and 3,0 Hz included. Delta power band was expressed in normalized units defined as the ratio between the power value within the specific frequency power band and the full night mean power value of the delta power band [47,48].

QRS complexes were automatically detected and an algorithm that we previously developed [42] computed RRI time series. Premature ventricular contractions and ectopic beats, as well as artifacts were removed and linearly interpolated with surrounding values in regards to the RRI time series if RRI < 350 msec or RRI > 1500 msec. RRI time series as well as all detected and interpolated events were visually inspected and corrected if necessary. RRI time series were resampled at 8 Hz. In accordance with the Task Force of the European Society of Cardiology and the North American Society of Pacing and Electrophysiology [33], we obtained cardiac spectral components of heart rate variability (HRV) every 20 seconds from a 120-seconds window that is shifted ahead 20 seconds by 20 seconds on the RRI time series [42]. We then obtained a power value for total frequency (TF) [0,01-0,4 Hz], very low frequency (VLF) (<0,03 Hz), low frequency (LF) [0,04-0,15 Hz] and high frequency (HF) [0,15-0,40 Hz] of HRV every 20 seconds. Thus, we reached synchronous power values every 20 seconds for EEG and ECG spectral components. HF or LF HRV powers are expressed in absolute (ms^2^) or normalized units (nu) that are the ratio between HF and the sum (HF + LF) [30,49]. In this sense, LF_nu_ = 1-HF_nu_. Values for LF_nu_ are not reported in the results. Cardiac sympatho-vagal influence is also described by the LF/HF ratio [35,49].

Data analysis as fast Fourier transform or HRV component calculations were computed with the software package Matlab and its signal processing toolbox (Matlab R2007b with Signal processing Toolbox 6.8, The Math Works Inc., USA).

A detailed description of recordings and data analysis can be found in a previous publication [42].

### Coherence analysis

The coherence analysis is a method that characterizes the linear relationship between two signals in the frequency domain [50]. In our study, we applied this method to measure the link between HF_nu_, which represents the cardiac vagal predominance of HRV, and delta power band, which is related to deep sleep. In regard to the stationary requirement for applying this method, we limited our analysis to the three first NREM-REM cycles [42].

At the frequency of the cross-spectrum maximum between HF_nu_ and delta signals, *f*_*NREM-REM*_, we calculated coherence, gain, phase shift and the delay values between the fluctuations in HF_nu_ and the fluctuations in the delta power band. The frequency of the main peak of cross-spectrum oscillations was around a rhythm of a sleep NREM-REM cycle and was defined below 1,1 × 10^-3^ Hz. This limit corresponds to the minimal duration between two successive REM epochs to define a new NREM-REM cycle [46]. The coherence value could be interpreted as the linear part of a signal that is explained by the other signal. Values of coherence are between 0 and 1. Zero reflects the absence of a linear link between both signals while a coherence value of 1 suggests a very strong linear link between HF_nu_ and delta signals. The gain value is the ratio between amplitude of both signals. The phase shift is the angular phase, expressed in degrees, while the delay is the angular phase divided by the frequency *f*_*NREM-REM*_ and converted to minutes. This indicates the duration between the occurrence of modifications in both signals.

### Statistics

All results are expressed as mean ± standard deviations. For the figures, standard errors were preferred to standard deviations. P values < 0,05 are considered as statistically significant. Variables for demographic, sleep and heart rate variability parameters as well as variables of coherence analysis between HF_nu_ and delta were compared between untreated patients suffering from SAHS and healthy controls and between CPAP treated patients and controls. Heart rate variability components were also compared between sleep stages in comparison to NREM sleep across the first three NREM-REM cycles.

Normality, homogeneity and homoscedasticity were tested for all groups of variables. If variables did not answer to the conditions, they were transformed to log_10_ or non-parametric tests were used. If all conditions were satisfied, general linear model (GLM) or T-Test for independent variables were used. To measure a potential effect of the body mass index (BMI) on cardiac autonomic components, bivariate correlations were applied between BMI values and each cardiac variable.

All the statistical analyses were computed with SPSS software (IBM SPPS Statistics, 20.0.0, USA).

## Results

### Demographic parameters

Eight untreated patients and the same eight patients treated with CPAP did not differ from fourteen healthy controls in terms of age, systolic blood pressure, and nicotine, alcohol or caffeine consumptions. BMI was higher in both groups of patients and diastolic blood pressure was increased only in patients receiving CPAP compared to controls. Mean arterial blood oxygen saturation (SaO_2_) was decreased in untreated apneic patients compared to healthy men. Bivariate correlations showed BMI to be unrelated to HRV components. Table 1 reports mean values.

**Table 1:** Demographic parameters

	**SAHS patients**	**SAHS patients**	**Healthy controls**
	**before CPAP**	**with CPAP**	
*N*	8	8	14
Age (years)	45,6 ± 6,7	49,9 ± 7	44,0 ± 6,2
BMI (kg/m^2^)	30,45 ± 5,75*	31,87 ± 4,5*	24,13 ± 2,34
SBP (mmHg)	110,0 ± 22,7	120 ± 30,7	118,2 ± 11,7
DBP (mmHg)	90,0 ± 31,6	99,3 ± 24,9*	70,4 ± 8,9
Nicotine (cig/day)	8,13 ± 10,33	0,00 ± 0,00	2,64 ± 7,19
Alcohol (U/day)	0,75 ± 1,66	0,67 ± 1,16	0,36 ± 0,6
Caffeine (100 mg/day)	2,63 ± 2,05	3,00 ± 0,00	2,72 ± 2,05
AHI (e^vts^/h)	63,50 ± 22,89***	5,50 ± 4,38	3,79 ± 3,17
*OAI (e*^*vts*^*/h)*	16,90 ± 12,38***	1,37 ± 2,15	0,88 ± 1,10
*CAI (e*^*vts*^*/h)*	6,65 ± 5,71**	0,60 ± 1,45	0,72 ± 1,24
*MAI (e*^*vts*^*/h)*	22,21 ± 21,43***	0,1 ± 0,28	0,02 ± 0,08
*HypoI (e*^*vts*^*/h)*	17,51 ± 9,22***	3,48 ± 2,50	2,21 ± 1,63
Mean SaO_2_ (%)	87,88 ± 5,36*	92,50 ± 2,00	93,19 ± 1,47
Bilateral PLMI (e^vts^/h)	11,83 ± 19,08	2,36 ± 2,38	0,59 ± 0,91

*SAHS*, Sleep apnea-hypopnea syndrome; *CPAP*, Continuous positive airway pressure; *BMI*, Body mass index; *SBP*, Systolic blood pressure; *DBP*, Diastolic blood pressure; *cig*, Cigarettes; *U*, Units; *AHI*, Apnea-hypopnea index; *OAI*, Obstructive apnea index; *CAI*, Central apnea index; *MAI*, Mixed apnea index; *HypoI*, Hypopnea index; *e*^*vts*^*/h*, Events per hour; *SaO*_*2*_, Mean arterial blood oxygen saturation; *PLMI*, Periodic leg movement index. Results expressed as mean ± standard deviation. * *P* < 0,05, ** *P* < 0,01, *** *P* < 0,001 vs. healthy controls.

Patients suffering from severe SAHS showed large values for all sleep breathing events and increased BMI in comparison to healthy men (controls). Patients treated by CPAP had larger BMI and DBP than controls.

CPAP treatment decreased the apnea-hypopnea index from 63,5 ± 22,9 events/hour to 5,5 ± 4,4 events/hour in treated patients and normalized the mean SaO_2_. Mean treatment duration was 4 ± 3 years. Mean compliance duration for CPAP treatment was 6 ± 1 hours/night. The AHI was similar between treated patients and controls. Patients with CPAP showed an AHI between 0 and 12 events/h.

### Sleep parameters

During the entire night, patients suffering from SAHS showed decreased mean duration of REM sleep compared to controls. This difference disappeared with CPAP treatment. Other sleep parameters were similar between untreated patients and healthy men. All sleep parameters were similar between CPAP treated patients and controls.

Across the first three NREM-REM cycles, NREM, REM and wake after sleep onset (WASO) durations were similar between untreated apneic patients and controls as well as between apneic patients treated by CPAP and controls. Mean light sleep (stages 1 + 2) duration was increased in untreated patients in comparison to controls while in CPAP treated patients, light sleep duration was similar to healthy men. Mean deep sleep (stages 3 + 4) duration was not different among patients with SAHS, CPAP treated patients and healthy controls (Table 2).

**Table 2:** Sleep parameters for the entire night and the first three sleep cycles

	**SAHS patients**	**SAHS patients**	**Healthy controls**
	**before CPAP**	**with CPAP**	
***Parameters for entire night***			
TIB (min)	470 ± 36	471 ± 44	466 ± 56
SPT (min)	444 ± 56	446 ± 58	444 ± 64
TST (min)	397 ± 48	401 ± 55	414 ± 61
Sleep efficiency (%)	84 ± 8	85 ± 8	87 ± 5
Sleep latency (min)	19 ± 9	23 ± 9	16 ± 10
Amount of nocturnal awakenings	74 ± 56	38 ± 14	38 ± 16
Amount of sleep changes	231 ± 99	167 ± 57	218 ± 81
NREM duration (min)	337 ± 35	309 ± 58	322 ± 47
REM duration (min)	63 ± 28*	89 ± 11	91 ± 24
WASO duration (min)	44 ± 30	48 ± 33	30 ± 18
NREM duration (% SPT)	76 ± 8	69 ± 8	73 ± 3
*Deep sleep duration (% SPT)*	3 ± 4	4 ± 7	7 ± 6
REM duration (% SPT)	14 ± 6**	20 ± 5	21 ± 4
WASO duration (% SPT)	10 ± 7	10 ± 6	7 ± 4
***Parameters for the first three NREM-REM cycles***			
Sleep cycles duration (min)	331 ± 55	316 ± 60	296 ± 35
NREM duration (min)	253 ± 43	229 ± 55	221 ± 24
*Light sleep duration (min)*	237 ± 65*	216 ± 58	190 ± 30
*Deep sleep duration (min)*	16 ± 25	13 ± 20	31 ± 24
REM duration (min)	47 ± 25	63 ± 21	63 ± 19
WASO duration (min)	31 ± 24	25 ± 17	12 ± 6

*SAHS*, Sleep apnea-hypopnea syndrome; *CPAP*, Continuous positive airway pressure; *TIB*, Time in bed; *SPT*, Sleep period time; *TST*, Total sleep time; *NREM*, Non rapid eye movement sleep; *REM*, Rapid eye movement sleep; *WASO*, Wake after sleep onset; light sleep: sleep stages 1 + 2; deep sleep: sleep stages 3 + 4; min: minutes. Results were expressed as mean ± standard deviation. * P < 0,05, ** P < 0,01 vs. healthy controls.

Patients suffering from severe SAHS showed decreased overnight REM duration, expressed in minutes (min) or in percentage of the SPT (% SPT), and increased light sleep duration over the first three NREM-REM cycles, in comparison to healthy controls. All other sleep variables were similar between untreated apneic patients and controls as well as between CPAP treated apneic patients and controls.

### Heart rate variability parameters

Across the entire first three NREM-REM cycles, similar spectral cardiac components of HRV were observed between untreated apneic patients and controls as well as between patients treated by CPAP and controls. The LF/HF ratio and mean durations of RR intervals and HF HRV, expressed in absolute or normalized units, did not differ between untreated or CPAP treated patients and healthy controls. Nevertheless, in SAHS patients with and without CPAP, standard deviation of absolute HF of RRI was larger than the mean value (Table 3).

**Table 3:** Heart rate variability parameters

	**SAHS patients**	**SAHS patients**	**Healthy controls**
	**before CPAP**	**with CPAP**	
***Parameters for the first three sleep cycles***			
TF (ms^2^)	3806,73 ± 1965,05	1516,12 ± 1126,28	2740,10 ± 2006,97
VLF (ms^2^)	1991,91 ± 1128,84	722,10 ± 447,80	1264,10 ± 1154,44
LF (ms^2^)	1120,43 ± 499,85	522,31 ± 497,23	995,51 ± 629,67
HF (ms^2^)	589,40 ± 711,42	255,41 ± 326,06	407,04 ± 359,76
HF_nu_	29,63 ± 16,90	32,19 ± 16,57	26,96 ± 11,95
LF/HF	3,52 ± 2,62	3,14 ± 2,79	3,60 ± 2,30
RRI (sec)	0,95 ± 0,06	0,95 ± 0,07	1,00 ± 0,15
***Parameters for the first three sleep cycles across sleep stages***			
NREM RRI (sec)	0,95 ± 0,06	1,01 ± 0,07	1,01 ± 0,16
REM RRI (sec)	0,96 ± 0,11	1,02 ± 0,06	0,98 ± 0,13
WASO RRI (sec)	0,92 ± 0,06	0,97 ± 0,06	0,97 ± 0,15
NREM HF_nu_	29,71 ± 17,52	32,81 ± 17,63	27,23 ± 12,23
REM HF_nu_	29,86 ± 15,86	30,93 ± 13,87	25,84 ± 13,97
WASO HF_nu_	30,25 ± 15,50	28,22 ± 12,28	24,86 ± 8,93
NREM LF/HF	3,84 ± 3,06	3,13 ± 2,66	3,52 ± 2,36
REM LF/HF	2,18 ± 2,38	3,09 ± 3,09	4,13 ± 3,15
WASO LF/HF	1,88 ± 1,35	3,70 ± 3,92	3,93 ± 3,18

*SAHS*, Sleep apnea-hypopnea syndrome; *CPAP*, Continuous positive airway pressure; *TF*, Total power of heart rate variability (HRV); *VLF*, Very low frequency power of HRV; *LF*, Low frequency power of HRV; *HF*, High frequency power of HRV; *RRI*, RR-interval duration; *NREM*, Non rapid eye movement sleep; *REM*, Rapid eye movement sleep; *WASO*, Wake after sleep onset; *ms*, Milliseconds; *nu*, Normalized units. Results were expressed as mean ± standard deviation. Comparisons were performed with healthy men (controls).

Mean RR-interval durations, spectral components of RRI (expressed in absolute units, ms^2^, or normalized units) and the LF/HF ratio were similar between untreated patients and controls as well as between CPAP treated patients and controls throughout the night and sleep stages.

**Figure 1:**
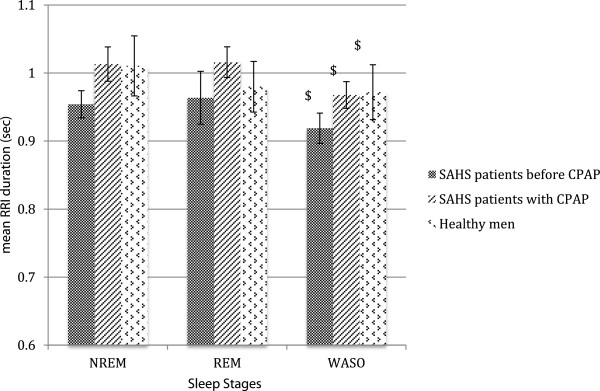
**Mean duration of RR intervals (RRI), expressed in seconds (sec), across sleep stages in SAHS patients before CPAP, with CPAP and healthy men (controls). **SAHS: sleep apnea-hypopnea syndrome; CPAP: Continuous positive airway pressure; NREM: non rapid eye movement sleep; REM: rapid eye movement sleep; WASO: wake after sleep onset. Mean values of the RR-interval durations were represented by boxes while standard errors were represented by bars in regard of three sleep stages. Each box colour was specific for each group of subjects. In each group, sleep stages (REM or WASO) were compared to NREM sleep. The symbol “$” correspond to a comparison between a specific sleep stage (REM or WASO) with NREM in a specific group of subjects with a P value < 0,001. RRI decreased from NREM sleep to wakefulness in each group of subjects.

Across sleep stages, RRI and cardiac spectral components of HRV as well as the LF/HF ratio did not differ between untreated patients and controls nor between CPAP treated patients and controls. For all groups, RRI was decreased during WASO in comparison to NREM sleep (Figure 1). Other cardiac parameters did not show significant differences between sleep stages and NREM sleep (Table 3).

In patients treated with CPAP, standard deviation values of the LF/HF ratio during REM and wake were at least as large as the mean values of LF/HF during the same sleep stages (Table 3).

### Coherence analysis

**Figure 2:**
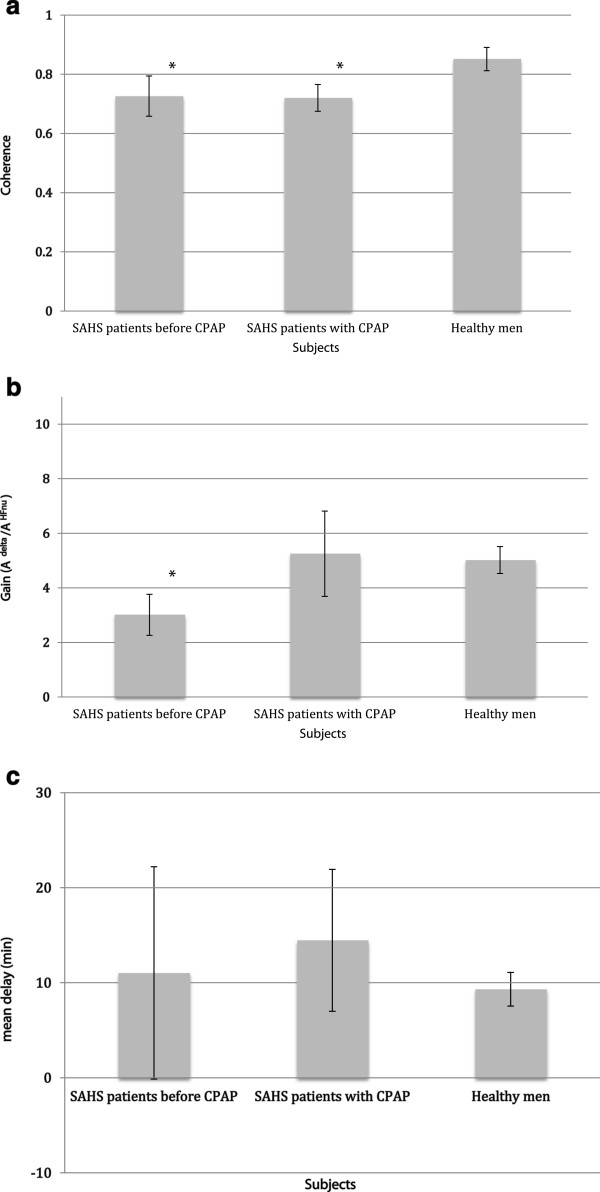
**Coherence analysis parameters between HF**_**nu **_**and delta power band signals.***Panel ****a****. *Mean coherence value in SAHS patients before CPAP, with CPAP and healthy men (controls). *Panel ****b****. *Mean gain value, expressed as the amplitudes’ ratio between delta power band and HF_nu _(A_delta_/A_HFnu_), in SAHS patients before CPAP, with CPAP and healthy men (controls). *Panel****c****. *Mean delay, expressed in minutes (min), between the occurrence of modifications in HF_nu _and those in delta power band in SAHS patients before CPAP, with CPAP and healthy men (controls). SAHS: sleep apnea-hypopnea syndrome; CPAP: Continuous positive airway pressure. For each panel, comparisons were performed with controls. Values were expressed as mean ± standard errors. ^*^ P < 0,05. Coherence values of the link between the relative cardiac vagal predominance and delta power band were decreased in untreated and long-term nasal CPAP treated apneic patients compared to controls. Gain values between these both signals decreased in untreated apneic patients in comparison to controls while during long-term nasal CPAP treatment, gain values were similar to controls. Delay between occurrence of modifications in HF_nu _and those in the delta power band did not differ between untreated apneic patients and controls or between CPAP treated patients and controls.

The frequency of the maximum of the cross-spectrum between HF_nu_ and delta, *f*_*NREM-REM*_, was decreased in untreated [(1,33 ± 0,25) 10^-4^ Hz] and treated patients [(1,34 ± 0,29) 10^-4^ Hz] compared to healthy controls vs [(1,87 ± 0,69) 10^-4^ Hz, *P* < 0,05 for each comparison]. Moreover, untreated patients showed decreased coherence and gain values in comparison to controls (Figure 2, *panel a, b*). During long-term CPAP treatment, gain values between HF_nu_ and delta signals were similar to controls while coherence values were decreased (Figure 2, *panel a, b*). Phase shift between occurrence of modifications in HF_nu_ and those in the delta power band did not differ between untreated apneic patients and controls (32,76 ± 85,70 vs. 33,44 ± 19,15 degrees) or between CPAP treated patients and controls (40,40 ± 56,63 vs. 33,44 ± 19,15 degrees). Similar results were obtained for the delay between occurrence of modifications in both signals and are shown in Figure 2, *panel c*. Additionally, standard deviations of the phase shift and delay were larger than the corresponding mean values for untreated and treated patients. In healthy controls, modifications occurrence in HF_nu_ preceded the occurrence of those in the delta power band with a phase advance corresponding to 9 ± 7 minutes.

## Discussion

Our study revealed that long-term use of nasal CPAP ameliorates some parameters reflecting the relationship between heart and brain during sleep in patients suffering from SAHS. Furthermore, in SAHS, the link between cardiac vagal influence and delta sleep is altered even in the absence of significant differences in mean spectral HRV components between apneic patients and healthy men.

Therefore, the permanence of altered parameters such as the tightness of the link related to coherence values, or such as the phase shift between modification occurrences in HF_nu_ and those in delta power bands, suggests that CPAP can not completely reverse the impairment of the relationship between heart and brain during sleep seen in patients suffering from SAHS. However, improved parameters of the relationship between cardiac sympatho-vagal balance and delta sleep, such as the gain between both signals, probably may contribute to the prevention of cardiovascular events in severely apneic patients treated with long-term CPAP.

Our patients suffering from severe SAHS showed decreased REM duration compared to healthy controls. Other sleep parameters did not differ from controls. Sleep characteristics of CPAP treated patients were similar to controls.

Our results were in accordance with the sleep architecture of patients suffering from moderate-to-severe SAHS, treated by an oral placebo versus by nasal CPAP for 6 to 12 months [18]. The authors of this study reported similar results between untreated and CPAP treated patients except a decreased deep sleep (sleep stages 3 + 4) in placebo patients. Sleep stage 1 duration and mean arousal index were increased in this latter group while CPAP treatment decreased these values [18]. In our sample, we observed that mean deep sleep duration was decreased in comparison to controls but this difference was not significant. Moreover, the lower REM duration observed in our untreated apneic patients, which increased with CPAP treatment to become similar to that of healthy controls, was also observed in apneic patients treated by placebo versus CPAP treatment without significant difference [18].

A previous study comparing sleep quality between sham-CPAP and effective-CPAP treatment in patients suffering from severe obstructive sleep apnea syndrome (OSAS) showed an absence of difference in sleep parameters between sham-CPAP and effective-CPAP treatment after seven days of treatment [51]. Nevertheless, a time effect was reported in both groups [51]. Our comparison between untreated patients and controls as well as between treated patients and controls confirmed the findings that CPAP did not ameliorate sleep parameters in comparison to healthy men with the exception of REM duration. In our study, we were not able to differentiate the role of CPAP treatment or the time effect of treatment.

Older publications showed increased REM duration as well as deep sleep after the first night of nasal CPAP treatment [17,52]. These changes were maintained after three days of CPAP treatment [52]. Observed values were compared with baseline and were not compared with healthy values. These increases were explained by a rebound effect [17,53]. Nevertheless, the authors did not determine precisely if these values were larger than those observed for healthy controls or if values became normal, as in healthy controls. In our study, we confirmed that patients suffering from severe SAHS showed shorter values of REM duration and that long-term nCPAP treatment restored REM duration to that of healthy controls.

In contrast with some data in the literature [11,12,54], our patients suffering from SAHS did not demonstrate significant changes of spectral cardiac components of HRV compared to healthy men. Nevertheless, our results were also in agreement with previous papers studying HRV components during rest [14], across sleep stages [16] or for normalized HRV components [12] in apneic patients.

Our SAHS patients treated by nCPAP did not differ significantly from healthy controls in terms of mean HRV components. Nevertheless, in treated SAHS compared to healthy subjects, there were strong trends towards lower absolute HRV values and increased HF_nu_ (hence lowered LF/HF). Indeed, assessment within sleep stages revealed that treated SAHS patients exhibited consistent trends towards greater HF_nu_ (lower LF/HF ratio) in each sleep state. Thus, we hypothesize that long-term nCPAP use could increase the cardiac vagal predominance of HRV.

Only a few studies evaluated the impact of long-term CPAP treatment on cardiac sympatho-vagal activity [19,20,25,26]. In patients suffering from OSA and heart failure, after one month of nCPAP, HF HRV, in absolute and normalized units, was increased during wakefulness in the morning compared to controls. Additionally, the LF/HF ratio was decreased [20]. During the night after three months of nasal CPAP treatment, LF/HF, VLF, LF, and LF_nu_ values were decreased while HF_nu_ was increased compared to patients with moderate-to-severe SAHS before treatment [19]. In contrast to the increased cardiac influence, the Chrysostomakis’group found decreased vagal tone after two months of CPAP treatment [25]. However, in his group of patients suffering from severe obstructive SAHS, vagal activity was increased during the night in comparison to healthy controls. CPAP treatment produced similar cardiac vagal activity to that of healthy controls [25]. After one, three and six months of CPAP therapy in moderate to severe SAHS patients, LF_nu_, HF_nu_, and the LF/HF ratio during spontaneous and deep breathing were similar to baseline values implying the absence of increased relative cardiac vagal predominance induced by CPAP treatment [26].

Similar controversy was reported during the first days of CPAP treatment use in SAHS patients [27,28,55]. Overnight LF and HF power values did not show differences between moderately apneic patients, severely apneic patients, CPAP treated patients, and healthy controls after one night of treatment [27]. In this study, patient groups were small (5 to 8 per group) and many had cardiac comorbidities and were treated by cardiotropic medications. Moreover, the standard deviation of LF/HF during NREM for moderate OSA and CPAP treatment was almost similar to the mean values. This indicates a large variability of cardiac sympatho-vagal balance between all four subjects of each group. Although that mean HR during the night as well as across sleep stages (NREM, REM, awake) was not different before and after one night of CPAP treatment in patients suffering from severe SAHS, HRV was decreased with the use of CPAP [28]. After 11 days of nCPAP treatment, the LF/HF ratio was lower at rest in the morning than the values observed after one and three nights of treatment as well as the baseline values before CPAP treatment [55]. Additionally, in the CPAP treated group, HF was increased beginning on the third day in comparison to the placebo group [55]. These results indicated that cardiac sympatho-vagal fluctuations were decreased in untreated apneic patients with lower vagal tone and that CPAP treatment increased cardiac vagal influence [55].

Thus, numerous studies reveal that vagal predominance of the cardiac sympatho-vagal balance was induced by CPAP treatment in comparison to severely apneic patients before treatment [19,20,55] but these results were controversial [26-28]. Moreover, only a few studies compared their results to healthy controls [25,27]. In our results, cardiac sympatho-vagal activity of CPAP treated patients did not differ from that of healthy men. However, HF_nu_, which reflects cardiac vagal predominance, tended to be greater compared to healthy controls without reaching statistical significance, a trend that was apparent whether we assessed HRV within individual sleep states or assessed the first 3 cycles of the night as a whole.

During the first three NREM-REM cycles, the LF/HF ratio did not differ from one in CPAP treated patients. This result indicates that none of the sympathetic and vagal branches of cardiac sympatho-vagal activity predominated, in contrast to the relative sympatho-vagal influence in healthy controls. Moreover, the absence of relative sympathetic or vagal predominance is found during REM sleep and wakefulness in patients treated by CPAP. This could imply that CPAP treatment increased cardiac vagal activity in contrast to the relative sympathetic activity. Indeed, CPAP treatment improved baroreflex sensitivity in SAHS [56,57]. During SAHS, chemoreflex sensitivity is increased and is associated with increased relative sympathetic predominance, while CPAP treatment could normalize the sympathetic response by decreasing sensitivity and tonic activity of the chemoreflex [9,58,59]. Moreover, readjusted chemosensitivity during CPAP treatment could increase baroreflex activity [58]. Then, long-term CPAP treatment could abolish the relative sympathetic predominance and increase the relative vagal influence of the cardiac sympatho-vagal balance during sleep.

In accordance with previous reports [16], our patients suffering from severe SAHS demonstrated decreased coherence and gain values compared to healthy men. The phase shift or the delay between occurrence of modifications in HF_nu_ and occurrence of those in the delta power band did not differ from controls, but showed larger fluctuations around the mean and did not certainly differ from zero. In agreement with our previous paper [16], SAHS altered the tightness of the link between cardiac sympatho-vagal influence and delta during sleep as well as the phase shift or the delay between occurrences of modifications in HF_nu_ and those in delta power band. This may be due to a more irregular breathing. In patients treated with long-term nCPAP, we observed decreased coherence and similar gain values in comparison to healthy controls. Moreover, the variability of the phase shift or the delay between HF_nu_ and delta modifications was decreased in comparison with untreated apneic patients, but phase shift and delay were not different from zero.

Thus, long-term nCPAP increases the strength of the interaction between the relative cardiac vagal predominance of HRV and delta sleep EEG but does not enhance the tightness of this link. CPAP treatment could ameliorate the quality of the information between brainstem cardiovascular and sleep structures through a regularisation of breathing and, as a result, an increase in the gain values and also a decrease in the variability of the time delay between modifications in cardiac sympatho-vagal influence and delta power band during sleep. Other non-linear biomathematical models that are specifically developed to determine the exact phase shift and the information flow of ECG-EEG signals, as phase slope index or Granger causality, could be applied to confirm our results [60].

Decreased frequency of the maximum of the cross-spectrum between HF_nu_ and delta indicated a lengthened mean sleep cycle duration and that the time at which both signals shared a maximum of power was later than that of healthy controls. Then, mean delay between modifications occurrence of HF_nu_ and delta signals was later in untreated and treated patients in comparison to healthy men.

Our patients were controlled with healthy men for age, nicotine, alcohol and caffeine consumptions. Moreover, they did not suffer from other pathologies than severe SAHS. They had no present or past medical condition that would modify cardiovascular or cerebral activities. They took no illicit drugs, psychotropic or somatic medications, or over the counter agents that could interfere with cardiac activity or sleep. Presumably, this “pure” sample does not reflect the general middle-aged population. The results are also limited by the small size of our groups. On the other hand, we can also ensure that comorbidities did not influence our analysis of relationship between cardiac vagal influence and delta sleep in SAHS and its adequate CPAP treatment. Nevertheless, we were not able to match BMI for patients and controls. The influence of BMI on HRV is controversial [61,62]. Using bivariate correlations, we concluded that BMI did not influence our HRV components. Additionally, our results were in accordance with those of the Kufoy and his group [28]. He studied HRV components in severe apneic patients with class I obesity before and after CPAP treatment [28]. Due to our sample size and their characteristics, our results should be confirmed by large population studies and further, tested on populations with comorbidities.

## Conclusions

Our data confirm alterations of the relationship between cardiac sympatho-vagal activity and delta power in patients suffering from severe SAHS, even in the absence of cardiovascular events or significant changes in HRV components. Moreover, new findings of this study reveal that long-term nCPAP treatment could ameliorate the interaction between heart and brain during sleep, and thus, avoid cardiovascular pathologies, even if CPAP treatment does not cure SAHS.

## Competing interests

The authors declared that they have no competing interest.

## Authors’ contributions

FJ is the principal investigator of the study and participated to each step of this research. CK performed statistics. JL provided his help for the data collection and the interpretation of the results. AN, PV and PL contributed to the study design and the interpretation of the results. All authors read and approved the final manuscript.

## Funding

Research reported in this paper was supported by the Belgian National Fund for Scientific Research and the Erasme Academic Hospital – ULB.

## Pre-publication history

The pre-publication history for this paper can be accessed here:

http://www.biomedcentral.com/1471-2466/13/29/prepub
